# Mini-Cog versus Codex (cognitive disorders examination) Is there a difference?

**DOI:** 10.1590/1980-57642020dn14-020005

**Published:** 2020

**Authors:** Andrew J. Larner

**Affiliations:** 1 Consultant Neurologist. Cognitive Function Clinic, Walton Centre for Neurology and Neurosurgery, Liverpool, United Kingdom.

**Keywords:** codex, dementia, Mini-Cog, mild cognitive impairment, sensitivity and specificity, codex, demência, Mini-Cog, comprometimento cognitivo leve, sensibilidade e especificidade

## Abstract

Mini-Cog and Codex (cognitive disorders examination) are brief cognitive screening tests incorporating word-recall and clock drawing tests.

**Objective:**

To assess and compare the screening accuracy of Mini-Cog and Codex for diagnosis of dementia and mild cognitive impairment (MCI) in patients attending a dedicated cognitive disorders clinic.

**Methods:**

Tests were administered to a consecutive cohort of 162 patients, whose reference standard diagnoses based on clinical diagnostic criteria were dementia (44), MCI (26), and subjective memory complaint (92).

**Results:**

Both Mini-Cog and Codex had high sensitivity (>0.8) for dementia diagnosis, but Codex was more specific. For diagnosis of MCI, Mini-Cog had better sensitivity than Codex. Weighted comparisons of Mini-Cog and Codex showed only marginal net benefit for Mini-Cog for dementia diagnosis but larger net benefit for MCI diagnosis.

**Conclusion:**

In this pragmatic study both Mini-Cog and Codex were accurate brief screening tests for dementia but Mini-Cog was better for identification of MCI.

## Resumo.

Mini-Cog e Codex são testes breves de triagem cognitiva incorporando testes de recuperação de palavras e desenho de relógio.

**Objetivo:**

Avaliar e comparar a precisão da triagem de Mini-Cog e Codex (exame de distúrbios cognitivos) para o diagnóstico de demência e comprometimento cognitivo leve (CCL) em pacientes atendidos em uma clínica dedicada a distúrbios cognitivos.

**Métodos:**

Os testes foram administrados a uma coorte consecutiva de 162 pacientes, cujos diagnósticos padrão de referência com base em critérios clínicos de diagnóstico foram demência (44), CCL (26) e queixa subjetiva de memória (92).

**Resultados:**

O Mini-Cog e o Codex apresentaram alta sensibilidade (>0,8) para o diagnóstico de demência, mas o Codex foi mais específico. Para o diagnóstico de CCL, o Mini-Cog teve melhor sensibilidade que o Codex. As comparações ponderadas de Mini-Cog e Codex mostraram apenas um benefício líquido marginal para o Mini-Cog para o diagnóstico de demência, mas um benefício líquido maior para o diagnóstico de CCL.

**Conclusão:**

Neste estudo pragmático, o Mini-Cog e o Codex foram testes breves de triagem precisos para demência, mas o Mini-Cog foi melhor para a identificação de CCL.

The assessment of patients with memory and other cognitive symptoms usually involves the administration of a cognitive screening instrument (CSI). Because consultations are often time limited, a number of brief CSIs which can be administered in less than 5 minutes have been developed, some derived from elements of the Mini-Mental State Examination (MMSE)^1^ and some based on clock drawing.^2^ Mini-Cog and Codex are examples of such brief CSIs.

Mini-Cog consists of a three word recall task and a clock drawing task.^3^ In the standard scoring system, a score of zero or three on the word recall task leads to categorization as “dementia” or “no dementia”; for the intermediate scores on word recall, 1 or 2, performance on the clock drawing task is then taken into account: if normal or abnormal the patient is categorized as “no dementia” or “dementia”, respectively.

**Figure 1:**
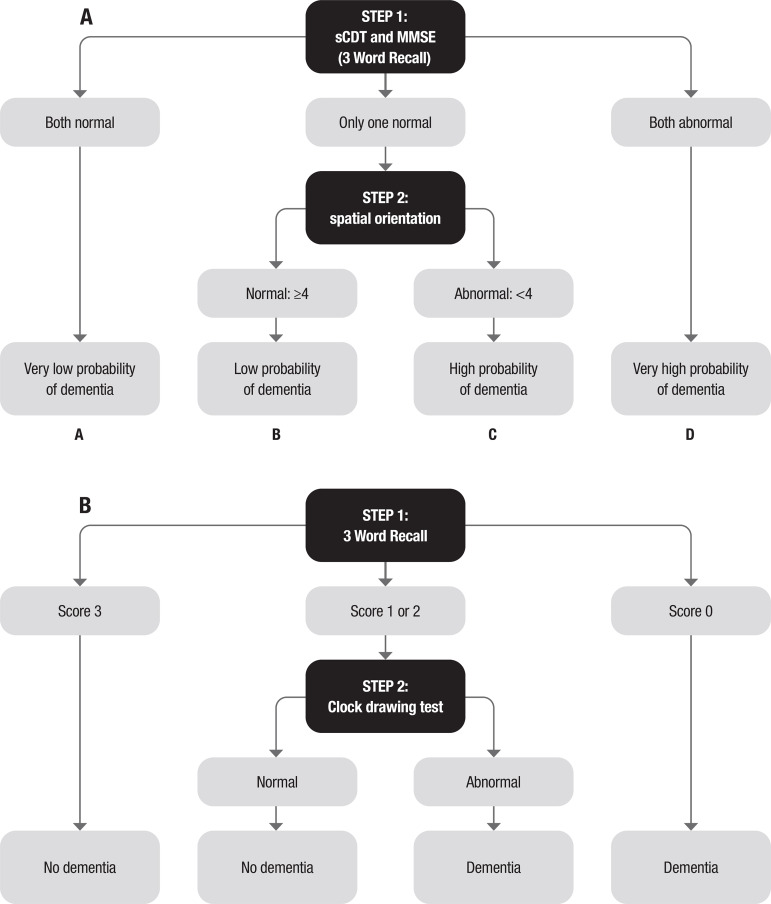
[A] Codex decision tree. [B] Mini-Cog rewritten as a decision tree using scoring method of Borson et al.^3^

The cognitive disorders examination or Codex is a two-step decision tree for diagnostic prediction which incorporates the three-word recall and spatial orientation components from the MMSE along with a simplified clock drawing test (CDT).^4^^,^ 5 The endpoint values of the four terminal nodes have different probabilities of dementia diagnosis: categories A-D, respectively with very low, low, high, and very high probability of dementia (Figure 1A).

There is item overlap between Mini-Cog and Codex. Rewriting Mini-Cog as a decision tree (Figure 1B) and comparing this with Codex, it is evident that the two sequential steps of Mini-Cog are applied simultaneously in Step 1 of Codex (Figure 1).

Do Mini-Cog and Codex perform similarly in clinical practice? The primary aim of this study was to compare the accuracy of these two CSIs in screening for dementia, a comparison which has not, to the best of the author’s knowledge, been reported hitherto in the literature. The secondary, exploratory, aim was to compare accuracy in screening for mild cognitive impairment (MCI), an important clinical distinction, although it is acknowledged that neither of these CSIs was specifically designed for the identification of MCI.

## METHODS

### Participants

Data from a consecutive patient cohort referred to a dedicated cognitive function clinic based in a secondary care setting (neurology clinic) over a fixed time period of nine months (February-November 2012 inclusive)^6^^,^ 7 and who were administered MMSE and CDT were re-analysed. Criterion diagnoses were dementia, mild cognitive impairment (MCI), or subjective memory complaint (SMC) by judgment of an experienced clinician, the former two diagnoses based on standard diagnostic criteria for dementia (DSM-IV; study data collection preceded publication of DSM-5) and MCI.^8^ Subjects gave informed consent and the study protocol was approved by the institute’s committee on human research.

### Procedures

For Mini-Cog, categorical data (dementia/no dementia) were derived using the standard scoring method (Figure 1B).^3^ For Codex, categorical data were derived from the decision tree (Figure 1A) with differing probabilities of diagnosis (A = very low, B = low, C = high, D = very high); categories C and D were taken to be indicators of cognitive impairment, as per the index publication.^4^ Neither Mini-Cog nor Codex categories were used in reference diagnosis to avoid review bias.

### Statistical analysis

STARDdem guidelines for reporting diagnostic test accuracy studies in dementia were observed.^9^ Standard summary measures of discrimination were calculated: sensitivity and specificity, Youden index (Y), positive and negative predictive values (PPV, NPV), predictive summary index (PSI), correct classification accuracy (Acc), net reclassification improvement (NRI), positive and negative likelihood ratios (LR+, LR-; classified according to Jaeschke et al.),^10^ diagnostic odds ratio (DOR), and clinical utility indexes (CUI+, CUI-; classified according to Mitchell).^11^ In addition, a number of recently described unitary metrics^12^ were calculated: the “likelihood to be diagnosed or misdiagnosed” which is the ratio of the number needed to misdiagnose (NNM = 1/(1 - Acc)) to either the number needed to diagnose (NND = 1/Y) or the number needed to predict (NNP = 1/PSI), where LDM >1 is desirable;^6^^,^ 12 and the summary utility index (SUI = CUI+ + CUI-) and the number needed for screening utility (NNSU = 1/SUI), with values classified as previously reported.^12^^,^ 13

Mini-Cog and Codex were compared by means of weighted comparison performed as per the method of Moons et al.:^14^

$$\mathrm{WC}=∆\;\mathrm{sens}+\left[\left(1-\pi /\pi \right)\times \mathrm{relative}\;\cos t\left(\mathrm{FP}/\mathrm{TP}\right)\times ∆\;\mathrm{spec}\right]$$

where π = prevalence; FP = false positives; and TP = true positives. Δ sens and Δ spec are the differences in the sensitivity and specificity of the two tests at the chosen test cut-offs. The relative misclassification cost (FP/TP), a parameter which seeks to define how many false positives a true positive is worth, was set at FP/TP = 0.1, following previous usage,^15^ and reflecting the clinical desire for high test sensitivity. From the WC values, equivalent increase (EI) in TP patients per 1000 tested was also calculated, using the equation:

EI=WC×prevalence×1000

## RESULTS

Baseline demographic data from the study are shown in Table 1, along with the distribution of observed Mini-Cog and Codex categories. Final diagnoses in the dementia group were Alzheimer’s disease or mixed Alzheimer’s disease and cerebrovascular disease (31), frontotemporal dementia (6), dementia with Lewy bodies (4), progressive supranuclear palsy (2) and alcohol-related dementia (1). In the MCI group, final diagnoses were amnestic MCI (19; single domain 8, multiple domain 11) and non-amnestic MCI (7; single domain 1, multiple domain 6).

**Table 1:** Study demographics and base category data.

Gender F:M (% female)	Age range (median)	Diagnosis: Dementia/MCI/SMC (%)	Mini-Cog category			Codex category			
			NoD (%)	Dem (%)		A (%)	B (%)	C (%)	D (%)
79:83 (49)	20-89 (61)	44/26/92 (27/16/57)	92 (56.8)	70 (43.2)		42 (25.9)	63 (38.8)	5 (3.1)	52 (32.1)

MCI: mild cognitive impairment; SMC: subjective memory complaint; NoD: no dementia; Dem: dementia.

Patient numbers with positive or negative Mini-Cog and Codex tests as a function of the reference diagnosis are shown in Table 2.

**Table 2:** Patient categorization by Mini-Cog and Codex as a function of the reference diagnosis.

Test: reference diagnosis	N	TP	FP	FN	TN
Codex: Dementia vs no dementia	162	37	20	7	98
Codex: MCI vs SMC	118	11	9	15	83
Mini-Cog: Dementia vs no dementia	162	39	31	5	87
Mini-Cog: MCI vs SMC	118	18	13	8	79

MCI: mild cognitive impairment; SMC: subjective memory.

Examining the primary study aim, for the diagnosis of dementia versus no dementia the measures of discrimination were similar for Mini-Cog and Codex, with Mini-Cog slightly better for sensitivity, NPV, and LR-, but Codex was slightly better on all other measures, including the unitary measures (Table 3, left hand columns).

**Table 3:** Measures of discrimination (with 95% confidence intervals) for primary (diagnosis of dementia versus no dementia) and secondary (diagnosis of MCI versus subjective memory complaint [SMC]) study aims using Codex and Mini-Cog.

	Dementia vs no dementia (= MCI + SMC)		MCI vs SMC	
N	162 (44 vs 118)		118 (26 vs 92)	
Prevalence (P = pre-test probability)	Dementia 0.27		MCI 0.22	
Pre-test odds (= P/1 - P)	Dementia 0.37		MCI 0.28	
	**Codex**	**Mini-Cog**	**Codex**	**Mini-Cog**
Sensitivity (Sens)	0.84 (0.73-0.95)	0.89 (0.79-0.98)	0.42 (0.23-0.61)	0.69 (0.51-0.87)
Specificity (Spec)	0.83 (0.76-0.90)	0.74 (0.66-0.82)	0.90 (0.84-0.96)	0.86 (0.79-0.93)
Youden index (= Sens + Spec - 1)	0.67	0.62	0.32	0.55
Positive Predictive Value (PPV = post-test probability)	0.65 (0.53-0.77)	0.56 (0.44-0.67)	0.55 (0.33-0.77)	0.58 (0.41-0.75)
Negative Predictive Value (NPV)	0.93 (0.89-0.98)	0.95 (0.90-0.99)	0.85 (0.78-0.92)	0.91 (0.85-0.97)
Predictive Summary Index (= PPV + NPV - 1)	0.58	0.51	0.40	0.49
Correct classification accuracy (Acc)	0.83 (0.78-0.89)	0.78 (0.71-0.84)	0.80 (0.72-0.87)	0.82 (0.75-0.89)
Net Reclassification Improvement (NRI = Acc - P)	0.56	0.51	0.58	0.60
Positive Likelihood Ratio (LR+)	4.96 (3.29-7.49) = moderate	3.37 (2.45-4.65) = moderate	4.32 (2.01-9.30) = moderate	4.90 (2.78-8.62) = moderate
Negative Likelihood Ratio (LR-)	0.19 (0.13-0.29) = large	0.15 (0.11-0.21) = large	0.64 (0.30-1.38) = slight	0.36 (0.20-0.63) = moderate
Diagnostic Odds Ratio (= LR+/LR-)	25.9 (17.2-39.1)	21.9 (15.9-30.2)	6.76 (3.14-14.5)	13.7 (7.77-24.1)
Post-test odds (= pre-test odds x LR+)	Dementia 1.85	Dementia 1.25	MCI 1.21	MCI 1.37
Positive Clinical Utility Index (CUI+ = Sens x PPV)	0.55 = adequate	0.494 = adequate	0.23 = very poor	0.40 = poor
Negative Clinical Utility Index (CUI- = Spec x NPV)	0.78 = good	0.70 = good	0.76 = good	0.78 = good
LDM (= NNM/NND, NNM/NNP)	4.02, 3.48	2.81, 2.26	1.57, 1.97	3.09, 2.75
Summary Utility Index (SUI + CUI+ + CUI-)	1.33 = good	1.19 = adequate	0.99 = adequate	1.18 = adequate
Number needed for screening utility (NNSU = 1/SUI)	0.752 = good	0.840 = adequate	1.01 = adequate	0.847 = adequate

Examining the secondary study aim, for the diagnosis of MCI versus SMC, all the examined measures were better for Mini-Cog compared to Codex with the sole exception of specificity (Table 3, right hand columns). Both tests showed low sensitivity for differentiating MCI from SMC.

Weighted comparison showed a minuscule net benefit for Mini-Cog versus Codex for the primary study aim of dementia diagnosis, with less than 6 additional TP patients identified per 1000 screened (Table 4).

**Table 4:** Weighted comparison of Mini-Cog and Codex for diagnosis of dementia vs. no dementia (n=162) and MCI vs. subjective memory complaint (n=118).

Diagnosis Test	Dementia			MCI	
	Mini-Cog	Codex		Mini-Cog	Codex
Sensitivity	0.886	0.841		0.69	0.42
Specificity	0.737	0.831		0.86	0.90
Prevalence	0.272			0.22	
∆ Sens	0.045			0.27	
∆ Spec	-0.094			-0.04	
Weighted comparison	0.0198 = net benefit			0.26 = net benefit	
Equivalent increase (extra TP cases detected per 1000 tested)	5.40			56.3	

Weighted comparison for the secondary study aim of MCI diagnosis showed a larger net benefit for Mini-Cog versus Codex, resulting in an equivalent increase of around 56 extra MCI cases detected per 1000 tested (Table 4).

## DISCUSSION

Both Mini-Cog and the Codex decision tree are quick and easy to use. The study data showed that for the primary study aim of dementia diagnosis both Codex and Mini-Cog had good and similar metrics.

For the secondary study aim of MCI diagnosis, both tests were poor at differentiating MCI from SMC, an unsurprising finding since neither test was designed for this purpose, and as previously noted for Codex in this dataset^6^^,^ 7 and in independent studies of Codex.^4^^,^ 5 ^,^ 16 ^,^ 17 Mini-Cog appeared better than Codex for MCI diagnosis. Reasons for this disparity are evident from perusal of data in Table 1: Mini-Cog classified more patients as impaired (category “dementia” = 43.2%) than Codex (categories C and D = 35.2%) suggesting it has greater sensitivity for cognitive impairment than Codex, which evidently classifies some impaired patients as having a low probability of impairment (false negatives).

A previous attempt to circumvent the lack of sensitivity of Codex for cognitive impairment short of dementia by means of simple modifications of the decision tree proved unsuccessful.^7^ The application of a simple logical “And” rule in the first Codex step (also known as conjunctive combination, or “believe the negative”) might be anticipated to reduce sensitivity, NPV, and LR-, as observed with other applications of the “And rule”. Application of the “Or” rule (compensatory combination; “believe the positive”) may be advantageous for case finding as this approach generally improves sensitivity.^18^

Limitations of this study include use of clinical diagnostic criteria for dementia and MCI and the cross-sectional design which risks incorrect categorization of cases. Use of clinico-biological criteria incorporating imaging and CSF biomarkers (not available to this clinic) and longitudinal follow-up for delayed verification of diagnosis might circumvent these problems. The spectrum of final diagnoses reflects the selection bias encountered in a clinic based in a neurology centre, related to the relatively young age of the patient population (median 61 years), hence possibly limiting the generalizability of the findings; the diagnostic spectrum is likely to differ in studies undertaken in other secondary care settings such as old age psychiatry or geriatric medicine.

Deriving Mini-Cog scores retrospectively from lengthier tests has the potential to introduce bias, although this approach has been noted in previous Mini-Cog studies included in systematic reviews.^19^ This methodological limitation may limit the generalizability of findings since CSIs are not typically used in this way in clinical practice. In addition, the sample size in this study was relatively small, and no power calculation to estimate sample sizes was undertaken as the study was retrospective. However, the sample size fell within the normative ranges calculated as acceptable (25-400) for common research designs.^20^

When clinical assessments are time limited, Mini-Cog may be a possible option if the clinician wants to avoid false negative diagnoses of cognitive impairment, i.e. requires a high sensitivity test. Another very short screening test which, like Mini-Cog and Codex, incorporates recall and clock drawing is the Rapid Cognitive Screen,^21^ which may also be combined with simple categorical clinical signs which may indicate presence of cognitive impairment (Triple Test).^22^ However, the potential advantages of test brevity should be weighed against the evidence suggesting that CSI length (number of test items) correlates positively with measures of diagnostic accuracy.^23^ Other CSIs with slightly longer application times, around 10 minutes, may also be effective for the identification of MCI, particularly the Montreal Cognitive Assessment and the Mini-Addenbrooke’s Cognitive Examination^24^^,^ 25 and the Qmci screen.^26^
